# Target Sestrin2 to Rescue the Damaged Organ: Mechanistic Insight into Its Function

**DOI:** 10.1155/2021/8790369

**Published:** 2021-11-02

**Authors:** Moein Ala, Seyed Parsa Eftekhar

**Affiliations:** ^1^School of Medicine, Tehran University of Medical Sciences (TUMS), Tehran, Iran; ^2^Student Research Committee, Health Research Center, Babol University of Medical Sciences, Babol, Iran

## Abstract

Sestrin2 is a stress-inducible metabolic regulator and a conserved antioxidant protein which has been implicated in the pathogenesis of several diseases. Sestrin2 can protect against atherosclerosis, heart failure, hypertension, myocardial infarction, stroke, spinal cord injury neurodegeneration, nonalcoholic fatty liver disease (NAFLD), liver fibrosis, acute kidney injury (AKI), chronic kidney disease (CKD), and pulmonary inflammation. Oxidative stress and cellular damage signals can alter the expression of Sestrin2 to compensate for organ damage. Different stress signals such as those mediated by P53, Nrf2/ARE, HIF-1*α*, NF-*κ*B, JNK/c-Jun, and TGF-*β*/Smad signaling pathways can induce Sestrin2 expression. Subsequently, Sestrin2 activates Nrf2 and AMPK. Furthermore, Sestrin2 is a major negative regulator of mTORC1. Sestrin2 indirectly regulates the expression of several genes and reprograms intracellular signaling pathways to attenuate oxidative stress and modulate a large number of cellular events such as protein synthesis, cell energy homeostasis, mitochondrial biogenesis, autophagy, mitophagy, endoplasmic reticulum (ER) stress, apoptosis, fibrogenesis, and lipogenesis. Sestrin2 vigorously enhances M2 macrophage polarization, attenuates inflammation, and prevents cell death. These alterations in molecular and cellular levels improve the clinical presentation of several diseases. This review will shed light on the beneficial effects of Sestrin2 on several diseases with an emphasis on underlying pathophysiological effects.

## 1. Introduction

Sestrin2, encoded by SESN2 gene, is a stress-inducible metabolic regulator and a conserved antioxidant protein that protects cells against reactive oxygen species (ROS). In response to oxidative stress, P53 induces the expression of Sestrin2 to prevent further cellular damage [1, 2]. Furthermore, P53, in a Sestrin2-dependent manner, regulates major signaling pathways such as AMP-activated protein kinase (AMPK)/mammalian target of rapamycin complex 1 (mTORC1) and toll-like receptor (TLR) signaling pathways that are heavily involved in the regulation of several physiologic and pathologic processes such as metabolism, inflammation, and growth [2, 3]. However, recent studies have shown that Sestrin2 can function through P53-independent mechanisms [4]. Besides, it has been elucidated that nuclear factor erythroid 2-related factor 2 (Nrf2), a major transcription factor for several antioxidant genes, binds to antioxidant-response element (ARE) within SESN2 gene, thereby promoting the expression of Sestrin2 and exerting cytoprotective effects against oxidative stress [5].

Sestrin2 inhibits nicotinamide adenine dinucleotide phosphate (NADPH) oxidase to decrease oxidative stress [3]. Furthermore, it was reported that Sestrin2 can modulate the expression of *uncoupling protein 1* (*Ucp1*) through attenuation of oxidative stress [6]. Ucp1 regulates heat generation and energy expenditure; hence, Sestrin2 can mechanistically modulate metabolic diseases [6].

The expression of Sestrin2 significantly increases during inflammatory response to protect against oxidative stress and confine the progressive organ damage [7, 8]. Consistently, Sestrin2 knockout is associated with a burst of oxidative stress, more severe inflammatory response, and exacerbation of disease manifestations [7, 8]. Redistribution and increased expression of Sestrin2 act as endogenous defense mechanisms against organ damage [9]. Sestrin2 is also a major player for immune regulation [10]. In addition, it was shown that Sestrin2 is involved in the protective effects of numerous drugs [11–13]. The expression of Sestrin2 decreases in older age, and its expression is negatively correlated with frailty and health status during senescence [14].

Accumulating evidence indicates that Sestrin2 plays a crucial role in the pathogenesis of several diseases, and pharmacological interventions to target it possess therapeutic value [15–17]. These findings are useful in determining the prognosis of several diseases and open new horizons for new therapeutic approaches. Understanding the biologic function of Sestrin2 and identifying its interactions with a wide variety of signaling pathways contribute to finding proper targets for drug development. Furthermore, comprehensive insight into the function of Sestrin2 in different organs is necessary for proper pharmacological intervention. Herein, this review discusses the beneficial effects of Sestrin2 on several diseases such as atherosclerosis, heart failure, myocardial infarction, liver fibrosis, kidney diseases, neurodegeneration, and other diseases where evidence is abundant.

## 2. Sestrin2 in Cardiovascular Diseases

### 2.1. Atherosclerosis

Atherosclerosis is an age-related disease, and age is an independent risk factor for its occurrence [18]. Dyslipidemia, endothelial dysfunction, immune dysregulation, and chronic inflammation of vessels' wall have been implicated in the development and progression of atherosclerosis [19–22]. Oxidation of cellular proteins, DNA, and particularly lipids is a great contributor for atherosclerosis, and therapeutic approaches to alleviate oxidative stress have been suggested for the proper management of atherosclerosis [23]. Recent studies demonstrated that Sestrin2 can profoundly benefit cardiovascular health by preventing atherosclerosis, and several mechanisms have been proposed in this regard. Here, we explain how Sestrin2 increases during atherosclerosis to compensate and reverse the pathologic alterations [24].

Patients with carotid atherosclerotic plaques have significantly higher plasma levels of Sestrin2 compared with subjects without carotid plaques. Further, plasma Sestrin2 positively and independently correlates with the presence and severity of carotid plaques [25]. Similarly, Kishimoto et al. revealed that patients with coronary artery disease have markedly higher plasma levels of Sestrin2 compared with those without coronary artery disease. In addition, Sestrin2 level was significantly associated with the number of >50% stenotic segments and severity score [26]. Sestrin2 level of >16.0 ng/mL was significantly (95% confidence of interval (CI), odds ratio (OR) 1.79 (1.09-2.95)) associated with atherosclerosis [26].

Pigment epithelium-derived factor (PEDF) could inhibit mTORC1 and induce autophagy in human umbilical vein endothelial cells by activating the P53/Sestrin2 signaling pathway. Silencing P53 or Sestrin2 abrogated the effect of PEDF on endothelial cell autophagy [27]. PEDF also suppressed the inflammatory response of macrophages and stabilized atherosclerotic plaques [28]. Sestrin2, via inhibition of mTORC1, can induce autophagy in macrophages, thereby enhancing their M2 polarization [29]. Autophagy plays a pivotal role in the stability of atherosclerotic plaques. Inhibition of autophagy particularly in macrophages leads to increased oxidative stress and apoptosis in lesional macrophages and stimulates plaque necrosis [30].

Hwang et al. revealed that Sestrin2 knockdown leads to a burst of oxidative stress; uncontrolled release of several proinflammatory cytokines (such as interleukin 6 (IL6) and tumor necrosis factor *α* (TNF-*α*)) and chemokines (such as monocyte chemoattractant protein 1 (MCP-1)); endoplasmic reticulum stress (ER stress); and apoptosis of endothelial cells in the lipopolysaccharide- (LPS-) mediated inflammation model [31]. In LPS-mediated inflammation, Sestrin2 knockdown resulted in downregulation of AMPK and led to overactivation of nuclear factor kappa B (NF-*κ*B) and increased expression of cell adhesion molecules such as intercellular adhesion molecule 1 (ICAM1), vascular cell adhesion molecular 1 (VCAM1), and E-selectin in endothelial cells. Moreover, an AMPK agonist completely reversed the aforementioned effects of LPS on Sestrin2 knockdown endothelial cells [31]. Sestrin2, through an AMPK-dependent manner, restores endothelial nitric oxide synthase (eNOS) expression and improves nitric oxide (NO) production [32]. Dysfunctional and decreased expression of eNOS is a major driver of atherosclerosis, and therapeutic interventions to enhance eNOS expression or inhibit its uncoupling are protective against atherosclerosis [33].

**Figure 1:**
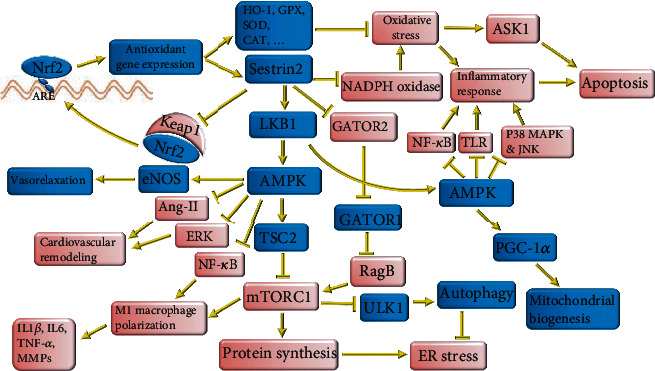
The molecular mechanisms underlying the protective effects of Sestrin2 on cardiovascular diseases. Sestrin2 releases Nrf2 through autophagic degradation of Keap1. Activated Nrf2 binds to ARE of antioxidant genes and promotes the expression of Sestrin2 and other antioxidants. Sestrin2 can inhibit NADHP oxidase. Attenuation of oxidative stress by Sestrin2 and other antioxidants suppresses the inflammatory response and attenuates ASK1-mediated apoptosis. Sestrin2 also activates the LKB1/AMPK/TSC2 pathway to inhibit mTROC1. Besides, Sestrin2 attenuates the inhibitory effect of GATOR2 on GATOR1, thereby inhibiting mTORC1. mTORC1 activation accelerates protein synthesis leading to deposition of a large amount of misfolded protein during inflammation and stimulating ER stress. Furthermore, mTORC1 activation downregulates autophagy and mitophagy by inhibiting ULK1 and other activators of autophagy. Sestrin2-induced inhibition of mTORC1 enhances autophagy and mitophagy and inhibits ER stress. AMPK activation by Sestrin2 improves mitochondrial biogenesis through upregulation of PGC-1*α*. AMPK inhibits Ang-II- and ERK-induced cardiovascular remodeling. Sestrin2 also attenuates oxidative stress and activates AMPK to inhibit TLR, NF-*κ*B, JNK, and P38 MAPK and confine the inflammatory response.

**Figure 2:**
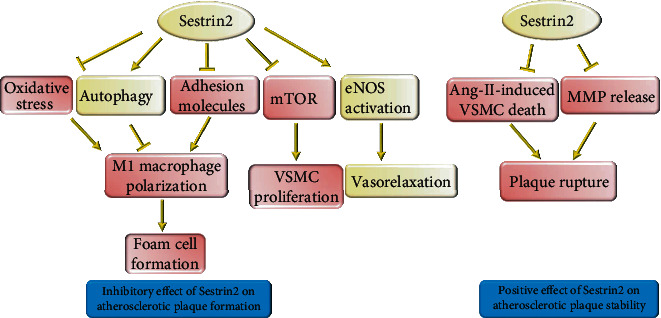
The protective effect of Sestrin2 on the formation and stability of atherosclerotic plaque. Sestrin2 attenuates oxidative stress and improves autophagy, thereby decreasing inflammation, downregulating the expression of adhesion molecules, and preventing M1 macrophage polarization and foam cell and plaque formation. Furthermore, Sestrin2 can stabilize the preexisting plaques by preventing VSMC apoptosis and MMP release.

Sestrin2 plays an important role in monocyte activation through the AMPK/mTORC1 pathway [34]. Sestrin2 activates AMPK, then it activates tuberous sclerosis complex 2 (TSC2) to inhibit mTORC1 [35]. In addition, Sestrin2 binds to GATOR2 to release GATOR1. Subsequently, GATOR1 binds to and inactivates RagB, a small GTPase essential for mTORC1 activation. Hence, in the presence of Sestrin2, GATOR1 can inhibit mTORC1 [36]. Moreover, Saxton et al. revealed that Sestrin2 is a leucine-sensitive regulator of mTORC1. In the presence of enough leucine amino acid, Sestrin2 will be released from GATOR2. Subsequently, GATOR2 attenuates the inhibitory effect of GATOR1 on mTORC1 (Figure 1) [37]. mTORC1 inhibition by Sestrin2 changes the balance between M1 and M2 macrophages, in favor of the M2 subtype. Overexpression of Sestrin2 in high glucose and dyslipidemic conditions could decrease CD80/CD68 and M1 macrophage-related cytokines such as IL6 and TNF-*α* and increase the release of M2 macrophage-related markers such as IL10 and CD163/CD68 [34]. Consistently, Sestrin2 overexpression hinders foam cell formation and prevents monocyte adhesion to endothelial cells [34]. Sestrin2 also decreases the release of matrix metalloproteinases (MMPs) such as MMP2 and MMP9, thereby enhancing the stability of atherosclerotic plaques [38]. Uncontrolled monocyte activation and foam cell formation are crucial for initial and later stages of atherosclerosis [39]. They lead to the production of a large amount of inflammatory cytokines as a major driver of atherosclerosis in the initial stages. In the later stages, increased expression of MMPs by these macrophages leads to the rupture of atherosclerotic plaques [39]. It was uncovered that inhibition of mTORC1 can decrease macrophage infiltration, inflammatory cytokine release, and lesion size in atherosclerosis [40]. Therefore, the inhibitory effect of Sestrin2 on foam cells can strongly protect against atherosclerosis (Figure 2).

Metabolic syndrome is associated with a markedly increased risk of atherosclerosis [41]. It was shown that Sestrin2 positively correlates with body mass index (BMI), waist circumference, high-sensitivity C-reactive protein (hs-CRP), and body fat percentage. Besides, Sestrin2 was significantly higher in patients with type 2 diabetes and metabolic syndrome [42, 43]. It seems that metabolic syndrome and AMPK regulate Sestrin2 in a feedback mechanism [42]. Furthermore, as an activator of AMPK, metformin increases Sestrin2 expression [34, 44], and the AMPK antagonist, compound C, decreases Sestrin2 expression [34]. In return, Sestrin2 activates AMPK, improves insulin resistance, and alleviates metabolic syndrome [45]. Oxidized lipoprotein stimulates Sestrin2 expression through the c-Jun N-terminal kinase (JNK)/c-Jun pathway. Sestrin2 upregulation attenuates oxidized lipoprotein-mediated ROS production and apoptotic cell death (Figures 1 and 2) [46].

Excessive proliferation and apoptosis of vascular smooth muscle cells (VSMCs) are involved in the progression of atherosclerosis and the rupture of atherosclerotic plaques, respectively [47]. Sestrin2 inhibits mTORC1 in VSMCs to suppress their excessive proliferation. Furthermore, Sestrin2 inhibits ROS production and prevents excessive apoptosis of VSMCs [48]. Likewise, it was shown that Sestrin2 can upregulate Nrf2 to protect VSMCs against Angiotensin-II- (Ang-II-) induced apoptosis (Figures 1 and 2) [49].

Endothelial progenitor cell (EPC) dysfunction is an important propellant of atherosclerosis. Ang-II induces apoptosis in EPCs and reduces Sestrin2 expression. Sestrin2 upregulation attenuates the deleterious effects of Ang-II on EPC and increases Nrf2 expression through P62-dependent autophagy [50]. In addition, Nrf2 silencing led to increased apoptosis of EPCs and EPC dysfunction through excessive ROS production [50]. Bae et al. uncovered that Sestrin2 can activate Nrf2, the chief regulator of antioxidant response, through autophagic degradation of Kelch-like ECH-associated protein 1 (Keap1) [51]. Releasing Nrf2 from Keap1 anchoring prevents its ubiquitination and subsequent proteolysis [51]. Nrf2, as a transcription factor, promotes the expression of several antioxidants such as Sestrin2 and heme oxygenase-1 (HO-1) by binding to their antioxidant response element (ARE) [5, 52, 53]. Nrf2 regulates physiological and pathophysiological processes in atherosclerosis, including lipid homeostasis, monocyte polarization, foam cell formation, and inflammation [54, 55]. Therefore, the mutual interaction between Nrf2 and Sestrin2 helps to maintain the protective function of Sestrin2 against oxidative stress (Figures 1 and 2).

### 2.2. Hypertension

Hypertension is a prevalent cardiovascular disease and consists of two subtypes, essential hypertension and secondary hypertension [56]. Essential hypertension is an age-related disease and constitutes 95% of hypertension cases [57]. Older age causes endothelial dysfunction due to oxidative stress and inflammation [58]. Additionally, increased renal ROS production plays a key role in the pathogenesis of hypertension [59]. Renal ROS activates the adrenergic system and the renin-angiotensin-aldosterone system (RAAS) and leads to water-salt retention [59]. Sestrin2 attenuates renal ROS and contributes to blood pressure regulation. Yang et al. showed that silencing Sestrin2 with siRNA increases renal ROS production, hyperoxidized peroxiredoxins, lipid peroxidation, and both systolic blood pressure and diastolic blood pressure in mice [60]. Moreover, they revealed that dopamine receptor D2 (D2R) depends on Sestrin2 to attenuate oxidative stress or lower blood pressure [60].

**Figure 3:**
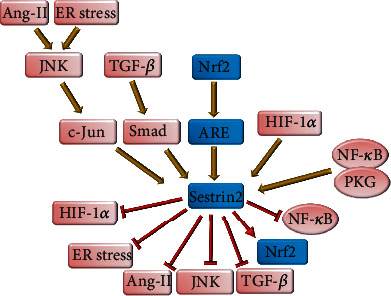
The autoregulatory effect of Sestrin2 on major signaling pathways. Several signaling pathways either protective such as Nrf2/ARE or destructive such as Ang-II, JNK/c-Jun, HIF-1*α*, NF-*κ*B, and TGF-*β*/Smad can induce Sestrin2 expression. In return, Sestrin2 selectively augments protective mechanisms and attenuates destructive signaling pathways.

Previously, it was mentioned that Sestrin2 can protect VSCMs against the deleterious effects of Ang-II [49]. Similarly, Sestrin2 can abrogate the detrimental effect of Ang-II on endothelial cells. Interestingly, Ang-II activates the JNK/c-Jun pathway to promote the expression of Sestrin2 (Figure 3). Subsequently, Sestrin2 attenuates Ang-II-induced oxidative stress and apoptosis in endothelial cells. Consistently, silencing Sestrin2 could enhance the harmful effects of Ang-II on endothelial cells (Figure 3) [24]. Furthermore, Sestrin2, in an AMPK-dependent manner, inhibits Ang-II-induced phosphorylation of myosin phosphatase target subunit 1 (MYPT1) and myosin light chain (MLC) in VSMCs to lower blood pressure [61]. AMPK activation can vigorously abrogate Ang-II-induced hypertension [61]. Hence, Sestrin2/AMPK can strongly protect against Ang-II-mediated hypertension. Furthermore, Sestrin2 can inhibit mTORC1 which is heavily involved in vascular remodeling [62].

Endothelial dysfunction is a major contributor for hypertension, atherosclerosis, and other cardiovascular diseases. Endothelial dysfunction impairs endothelial-dependent vasorelaxation and provokes inflammatory response [63]. As mentioned previously, Sestrin2 can enhance eNOS function leading to vasorelaxation and modulation of endothelial dysfunction [32].

It was explained that Sestrin2 can activate Nrf2, thereby enhancing the expression of itself and other antioxidants. Nrf2 is necessary for effective regulation of blood pressure, and Nrf2 deletion or inhibition increases blood pressure in mice [64, 65]. Nrf2 inhibition provokes oxidative stress, enhances inflammatory response, and exacerbates renal function [65]. Moreover, restoration of Nrf2 function alleviates renal inflammation, improves kidney function, and lowers blood pressure in rats [65, 66]. Nrf2 prevents redox hypertensive effects by activating the antioxidant genes such as superoxide dismutase 1 (SOD1), catalase (CAT), glutathione peroxidase (GPX), and thioredoxin-1,2 (TRDX-1,2) (Figure 1) [67].

### 2.3. Arrhythmia

ROS are strongly involved in the pathophysiology of atrial fibrillation (AF) [68]. It was shown that patients with AF have higher plasma levels of malondialdehyde (MDA) and oxidized low-density lipoprotein (ox-LDL) and lower levels of SOD, compared with healthy subjects [69]. Furthermore, these findings were more pronounced in patients with permanent AF, compared with patients with paroxysmal and persistent AF. In addition, plasma levels of ROS were positively correlated with the size of the left atrium [69]. Oxidative stress leads to calcium overload, mitochondrial damage, RAAS system activation, and dysfunction of nitric oxide synthase [70]. Also, oxidative stress is the reason for the higher incidence of AF among patients with chronic inflammatory diseases such as diabetes [71]. Oxidative stress stimulates atrial fibroblast proliferation, and inhibition of oxidative stress can prevent atrial remodeling [71, 72]. Mitochondrial ROS can alter the phosphorylation levels of major ion transport proteins, thereby leading to electrical instability, ventricular arrhythmia, and sudden cardiac death [73]. Oxidative stress activates calcium–calmodulin-dependent protein kinase II (CaMKII) which subsequently leads to early afterdepolarization- (EAD-) mediated AF [74]. CaMKII increases Ca^2+^ sparks and waves by phosphorylation of Na_V_1.5 in the atrium, leading to abnormal impulse generation [75]. Na_V_1.5 is a voltage-gated sodium channel subunit whose activation is associated with fast entry of Na^+^ into cardiomyocytes and results in multifocal atrial and ventricular ectopy [76]. Consistently, it was shown that CaMKII is upregulated in the early stages of AF, and inhibition of CaMKII can prevent atrial hypertrophy and fibrosis [77].

However, Sestrin2 cannot directly affect the heart's electrical function, but it can protect cardiomyocytes against oxidative stress and fibrosis [38, 78]. As mentioned, as a stimulator of cardiac remodeling, Ang-II changes the normal structure of atriums, particularly the left atrium, and induces AF [79]. Huang et al. showed that inhibition of Ang-II reduces the occurrence of AF in patients with hypertrophic cardiomyopathy [80]. Beyond its inhibitory effect on oxidative stress, Sestrin2 can suppress Ang-II-mediated alterations which protects against arrhythmias [24, 49].

### 2.4. Myocardial Ischemia-Reperfusion (IR)

However, reperfusion is considered the best solution to decrease the destructive effects of ischemia, and it has its own destructive effects known as ischemia-reperfusion injury (IRI) [81]. Studies suggest that several mechanisms such as impaired mitochondrial biogenesis, mitochondrial calcium overload, mitochondrial fission, inadequate mitochondrial ATP production, uncontrolled ROS production, autophagy dysfunction, and platelet activation are involved in the pathogenesis of IRI [82–84]. Moreover, free radicals and cytokines produced by white blood cells aggravate IRI by damaging endothelial cells [85]. ROS are mainly produced in the reperfusion phase of myocardial IR. They can directly damage cell membrane and impair intracellular calcium homeostasis leading to Ca^2+^ overload and increased permeability of the mitochondrial membrane, known as mitochondrial permeability transition. These cellular events finally result in cardiomyocyte necrosis [86]. Similarly, ROS can activate major inflammatory molecules such as NF-*κ*B and trigger the release of several inflammatory cytokines such as IL1*β*, IL6, and TNF-*α*. These cytokines activate apoptotic cell death during IRI [86]. Moreover, ROS are involved in cardiac remodeling after myocardial infarction [86]. It was shown that Sestrin2, through attenuation of oxidative stress, can inhibit the activation of JNK and P38 mitogen-activated protein kinase (P38 MAPK) and subsequently downregulate NF-*κ*B in myocardial IRI [87]. By attenuating NF-*κ*B signaling during myocardial IRI, Sestrin2 vigorously decreased the uncontrolled release of inflammatory cytokines such as IL1*β*, IL6, TNF-*α*, INF-*γ*, and IL17. These inflammatory cytokines, especially IL17, can cause a reinforcement cycle of the inflammatory response inhibited by Sestrin2 (Figure 1) [87]. Interestingly, Sestrin2 also prevented Ca^2+^-induced mitochondrial permeability transition and restored membrane potential during myocardial IRI [88, 89]. Sestrin2-mediated upregulation and activation of Nrf2 plays a pivotal role in attenuating oxidative stress in myocardial IRI [88, 89]. Consistently, Nrf2 inhibition causes myocardial IRI to deteriorate [88].

Previous studies showed that Sestrin2 can confine the destructive effect of IRI, particularly myocardial IRI, by reprograming intracellular signaling pathways. Quan et al. showed that Sestrin2, in an LKB1-dependent manner, activates AMPK, thereby improving glucose uptake and oxidation and limiting the size of the infarct zone in the mice model of myocardial IR (Figure 1) [90]. AMPK alleviates myocardial IRI through the prevention of mitochondrial ROS production and inhibition of the c-Jun N-terminal kinase (JNK) signaling pathway (Figure 1) [91]. In the absence of AMPK, overproduction of mitochondrial ROS and subsequent activation of JNK accelerate myocardial necrosis [91]. Morrison et al. observed that Sestrin2 expression increases in the early stages of myocardial ischemia and mitigates myocardial IRI through the LKB1/AMPK pathway. Sestrin2 knockout profoundly downregulated the LKB1/AMPK signaling pathway, increased infarct size, and impaired myocardial contractile function after IR [92]. Sestrin2 knockout is also associated with NF-*κ*B overactivity and increased expression of inflammatory cytokines such as IL1*β*, IL17A, TNF-*α*, and INF-*γ* in the myocardium [87]. By inhibiting mTORC1, Sestrin2 attenuates M1 macrophage-mediated inflammatory response after myocardial infarction and enhances heart function [93]. Furthermore, Sestrin2-mediated inhibition of mTORC1 was shown to prevent IRI-induced ER stress during myocardial IRI. Indeed, inhibition of mTORC1 hinders the accumulation of unfolded proteins (Figure 1) [94]. Augmentation of inflammatory response due to Sestrin2 knockout impairs myocardial recovery after IRI and mimics age-related myocardial susceptibility to IRI [87].

Sestrin2 improves myocardial mitochondrial biogenesis after IRI. Herein, Sestrin2 enhances the AMPK/PGC-1*α* pathway and increases the expression of Ucp1 and mitochondrial transcription factor A (TFAM) [95, 96]. Ucp1 regulates mitochondrial ATP and ROS production as well as heat generation [97]. TFAM modulates calcium homeostasis and mitochondrial ROS production to confine myocardial injury after IRI. Increased expression of Ucp1 and TFAM can vigorously alleviate myocardial IRI [96, 98]. Interestingly, it was revealed that Sestrin2 is necessary for the competent function of oxidative phosphorylation of mitochondrial complexes under physiologic condition or I/R stress [89]. In the absence of Sestrin2, mitochondrial complexes and the Krebs cycle cannot effectively produce ATP [89]. Increased expression of Sestrin2 during myocardial IRI is a compensatory mechanism to improve mitochondrial function and mitigate myocardial injury [89].

Mitophagy is a subtype of autophagy that helps to remove dysfunctional mitochondria and protect against myocardial IRI [99]. Kumar and Shaha showed that Sestrin2 stimulates unc-51-like kinase 1- (ULK1-) mediated phosphorylation of Beclin1 and potentiates the interaction between Beclin1 and Parkin. Subsequently, Parkin translocation on the mitochondrial surface activates mitophagy [100, 101].

### 2.5. Heart Failure and Cardiomyopathy

Oxidative stress has been introduced as the main driver of development and progression of heart failure. As mentioned previously, the increased amount of ROS impairs Ca^2+^ homeostasis and leads to contractile dysfunction [102]. ROS upregulate NF-*κ*B, P38 MAPK, and other signaling molecules to activate parallel inflammatory signals or activate apoptosis signal-regulating kinase 1 (ASK1) that leads to apoptosis (Figure 1) [102]. Furthermore, ROS stimulate fibroblast proliferation and production of excessive amount of MMPs [102]. Cardiac fibroblasts secrete extracellular matrix (ECM) to maintain the structure and function of the myocardium under oxidative stress, pressure overload, and chronic ischemia. Cardiac fibroblasts play a crucial role in cardiac fibrosis and remodeling [103]. It was shown that the salivary levels of ROS increase with the progression of heart failure [104]. Patients with CHF have higher plasma levels of Sestrin2. Sestrin2 plasma levels also positively correlate with disease severity in patients with CHF, and higher Sestrin2 levels predict increased incidence of major adverse cardiac events and poor outcome of the disease [105, 106]. Similarly, it was shown that Sestrin2 increases in the rat model of doxorubicin-induced cardiomyopathy [106]. Besides, Sestrin2 knockout led to increased fibrogenesis and development of more severe pressure overload cardiac remodeling and hypertrophy in rats [107]. Sestrin2 knockout disrupted mitochondrial function of cardiomyocytes and was associated with decreased glucose oxidation; decreased PGC-1*α* and mitochondrial DNA; and elevated levels of 4-hydroxynonenal, a lipid peroxidation byproduct, in the rat model of pressure overload-mediated heart failure [107]. Sestrin2 knockout decreased autophagy and increased apoptotic cell death of cardiomyocytes, as well [107].

Furthermore, Sestrin2 protects against cardiac hypertrophy by inhibiting mTORC1. mTORC1 phosphorylates and negatively regulates ULK1/2 and other autophagy-related molecules to inhibit autophagy (Figure 1) [107–110]. Age-related reduction in Sestrin2 has been proposed as a probable mechanism for cardiac dysfunction and remodeling in older age [109]. A sufficient amount of autophagy is pivotal for the removal of dysfunctional organelles and nonfunctional proteins and the prevention of apoptosis in the heart [111]. Likewise, insufficient autophagy is associated with related cardiomyopathy [111].

Dong et al. revealed that Sestrin2 prevents cardiomyocyte hypertrophy through the downregulation of the extracellular signal-regulated kinase 1/2 (ERK1/2) signaling pathway [112]. Prolonged activation of ERK causes maladaptive cardiac hypertrophy during pressure overload or anticancer chemotherapy (Figure 1) [113].

RAAS inhibitors can effectively prevent cardiac remodeling, decrease new-onset heart failure, reduce cardiovascular mortality and myocardial infarction, improve preexisting heart failure, and decrease cardiovascular death among patients with heart failure [114, 115]. Herein, Sestrin2 can attenuate the deleterious effect of Ang-II on cardiomyocytes and prevent Ang-II-mediated apoptosis of cardiomyocytes (Figure 1) [116].

It was shown that Sestrin2 and Sestrin2-mediated activation of Nrf2 can protect against pressure overload and obesity-induced cardiac remodeling in mice [11, 117]. Consistently, it was shown that Nrf2^−/−^ mice are more susceptible to cardiac remodeling, and Nrf2 is protective against pressure overload-mediated, ischemia-induced, diabetes-mediated, and anticancer drug-mediated cardiac remodeling [118]. For instance, it was shown that Nrf2 knockout mice rapidly develop cardiac hypertrophy and heart failure following myocardial IRI or diabetes [119, 120].

Empagliflozin, a sodium-glucose cotransporter 2 (SGLT-2) inhibitor, could mitigate obesity-induced cardiac dysfunction in mice [121]. It was shown that empagliflozin enhances the Sestrin2/AMPK/mTORC1 signaling pathway and augments the Nrf2/HO-1 pathway and eNOS activity to improve cardiac function [121]. In addition, the beneficial effects of empagliflozin were vigorously reversed in Sestrin2 knockout mice [121]. Interestingly, it was uncovered that SGLT-2 inhibitors can significantly decrease major adverse cardiovascular events, atherosclerotic cardiovascular disease, cardiovascular death and hospitalization because of heart failure, and progression of renal disease among diabetic patients [122].

Here, we explained how Sestrin2 is upregulated in several cardiovascular diseases to compensate for the damage. We also illustrated several mechanisms involved in the protective effects of Sestrin2 on cardiovascular diseases. Regarding these findings, the clinical application of Sestrin2 in cardiovascular diseases is worth investigating.

## 3. Sestrin2 in Liver Diseases

### 3.1. NAFLD

**Figure 4:**
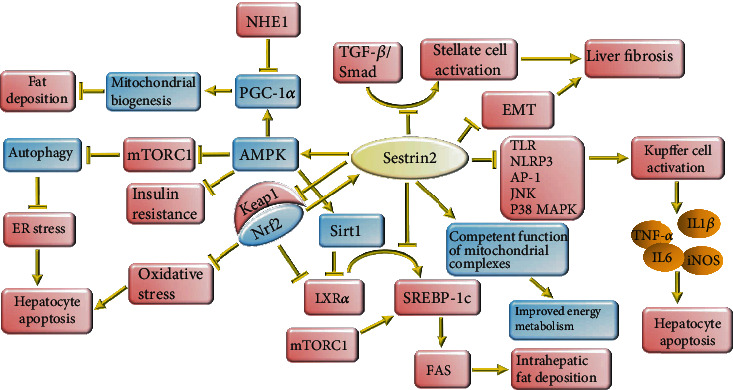
The protective effects of Sestrin2 on liver diseases. Similar to the cardiovascular system, Sestrin2 activates Nrf2 in the liver, thereby increasing the gene expression of itself and other antioxidants and attenuating oxidative stress. Also, Sestrin2 can improve insulin resistance, autophagy, mitophagy, and ER stress by activating the AMPK/mTORC1 axis. Upregulation of PGC-1*α* by Sestrin2 helps to improve mitochondrial biogenesis and prevent NHE1-mediated intrahepatic fat accumulation. Furthermore, Sestrin2 can prevent de novo lipogenesis by attenuating LXR*α*- and mTORC1-mediated SREBP-1c upregulation. Activation of Nrf2 and APMK/Sirt1 signaling also contributes to inhibiting LXR*α*-induced de novo lipogenesis. Sestrin2 is needed for competent function of mitochondrial complexes, and inadequate Sestrin2 expression impairs ATP production. Sestrin2 inhibits the major positive regulators of inflammatory signaling such as TLR, JNK, NLRP3, P38 MAPK, and AP-1 to prevent Kupffer cell activation and decrease cytokine release. Sestrin2 modulates TGF-*β*/Smad-mediated activation of stellate cells and hinders EMT to suppress liver fibrogenesis. These alterations alleviate liver inflammation, prevent intrahepatic fat deposition, and ameliorate liver fibrosis.

Oxidative stress and lipid oxidation have been implicated in the pathogenesis of NAFLD, as the most common liver disease [123]. Patients with NAFLD and nonalcoholic steatohepatitis (NASH) have higher levels of oxidative stress, and treatment with antioxidants can partly alleviate liver steatosis and inflammation [123]. Previous studies revealed that Sestrin2 protects against NAFLD and mediates the protective effects of different drugs on NAFLD. It was shown that carbon monoxide protects against hepatic steatosis by increasing Sestrin2 expression [124]. Sestrin2-mediated inhibition of mTORC1 attenuated hepatic ER stress, hepatic steatosis, and fibrosis in mice fed with a high-fat diet [125, 126]. Defective and insufficient autophagy response in patients with NASH or in mice fed with a high-fat diet increases ER stress and leads to apoptotic cell death of hepatocytes [127, 128]. Pharmacological activation of autophagy and inhibition of ER stress prevented apoptosis of hepatocytes and ameliorated NAFLD in mice fed with a high-fat diet (Figure 4) [129].

Previously, it was shown that glucagon-like peptide 1 (GLP1) agonists improved hepatic steatosis [130]. Liraglutide, a GLP1 agonist, increased the expression of Sestrin2 to protect against NAFLD. Sestrin2 activated the AMPK signaling pathway and augmented the Nrf2/HO-1 axis to attenuate oxidative stress and suppress the inflammatory response [131]. In addition, Sestrin2 knockout made hepatocytes susceptible to oxidative stress [51]. Sestrin2 binds to sequestosome-1 (SQSTM1) to accelerate autophagic degradation of Keap1 and activate Nrf2 [132]. SQSTM1 activates the AMPK/mTOR/ULK1 signaling pathway leading to autophagic degradation of Keap1 [132]. Nrf2^−/−^ decreases hepatic antioxidant expression, activates NF-*κ*B, pronouncedly aggravates liver inflammation, and is associated with more severe hepatic steatosis [133]. Hence, Sestrin2, through the activation of Nrf2, can partly increase the expression of itself and other antioxidants in the liver (Figure 4).

Increased expression of Sestrin2 has been associated with the repression of liver X receptor *α*- (LXR*α*-) mediated sterol regulatory element binding protein-1c (SREBP-1c) and fatty acid synthase (FAS) expression [12]. Liver X receptor-*α* (LXR*α*) activates de novo synthesis of fatty acids in the liver. It upregulates SREBP-1c to increase the target gene expression for lipogenesis [12]. mTORC1 also upregulates SREBP-1c to enhance lipogenesis [134]. Additionally, Nrf2 and AMPK/NAD-dependent protein deacetylase sirtuin1 (SIRT1) signaling can inhibit LXR*α* and suppress LXR*α*-mediated de novo lipogenesis in the liver [135]. Hence, Sestrin2 can mitigate NAFLD through direct or indirect downregulation of this major lipogenesis pathway (Figure 4).

Sestrin2 expression was shown to be negatively regulated by sodium-hydrogen exchanger 1 (NHE1). Indeed, a high-fat diet increased NHE1 expression which was associated with hepatic steatosis. NHE1 knockout increased the gene expression of Sestrin2 and PGC-1*α* and ameliorated insulin resistance [136]. Furthermore, it was shown that Sestrin2 promoted the expression of PGC-1*α* in liver cancer cells, and downregulation of Sestrin2 led to decreased expression of PGC-1*α* [137]. Interestingly, it was demonstrated that PGC-1*α* polymorphisms leading to its lower expression were contributory factors for the development of NAFLD [138]. It is probable that the improved function of Sestrin2 can attenuate the deleterious effects of NHE1 on PGC-1*α* and hepatic steatosis (Figure 4). Meanwhile, Sestrin2, in an AMPK-dependent manner, improves insulin resistance which finally results in decreased intrahepatic de novo lipogenesis and alleviates liver inflammation [45, 139].

### 3.2. Liver Fibrosis

In response to liver injury, liver macrophages known as Kupffer cells are the major source of ROS [140]. ROS produced by Kupffer cells induce necrosis and apoptosis of hepatocytes and stimulate hepatic stellate cells, leading to extracellular matrix deposition [140]. Therefore, inhibition of oxidative stress has been proposed as a therapeutic approach for liver fibrosis [140]. It was observed that patients with cirrhosis or mice with hepatic fibrosis have decreased expression of Sestrin2 in their liver [15, 141]. Sestrin2 inhibits hepatic stellate cell activation and prevents transforming growth factor *β*- (TGF-*β*-) mediated liver fibrosis (Figure 4) [15, 142]. Sestrin2 overexpression has been associated with decreased expression of collagen 1A1 and smooth muscle actin *α* (SMA-*α*) and reduced tissue level of inflammatory cytokines such as IL1*β* and TNF-*α* in the mice model of carbon tetrachloride- (CCl4-) induced liver fibrosis [142]. The TGF-*β* signaling pathway promotes the expression of profibrotic genes such as collagen fibers, plasminogen activator inhibitor-1 (PAI-1), and connective tissue growth factor (CTGF) through Smad 2 and 3. Smads are transcription factors that can promote specific gene expression [143]. Interestingly, the upstream region of the SESN2 gene possesses a putative Smad-binding element (SBE) sequence, and it was shown that during overactivity of the TGF-*β* signaling pathway, Smad increases Sestrin2 expression. Sestrin2 overexpression during the activation of the fibrogenic signaling of the TGF-*β*/Smad axis could protect against liver fibrosis, but deletion of SBE decreased the expression of Sestrin2 causing TGF-*β*-mediated liver fibrosis to deteriorate (Figure 3) [141]. Similarly, Sestrin2 overexpression was associated with reduced serum levels of alanine transaminase (ALT) and aspartate transaminase (AST) and prevented epithelial-mesenchymal transition (EMT) [142]. EMT expedites fibroblastic transformation of endothelial cells and progression of liver fibrosis [144]. Sestrin2 acts as a compensatory mechanism that is activated by the TGF-*β*/Smad signaling pathway and attempts to attenuate this signaling pathway. Exhaustion of Sestrin2 is usually found in the late stages of liver fibrosis [141].

Mice infected with the recombinant adenovirus SESN2 (Ad-SESN2) showed less severe hepatic injury in response to Gal/LPS-induced hepatic injury. Sestrin2 attenuated TLR-mediated inflammatory response and decreased the expression of inducible nitric oxide synthase (iNOS), IL1*β*, IL6, and TNF-*α* [3]. Sestrin2 profoundly ameliorates macrophage response by attenuating NLR family pyrin domain containing 3 (NLRP3) signaling and improving mitophagy (Figure 4) [145]. Sestrin2 could downregulate activator protein 1 (AP-1) [3]. AP-1 is a transcription factor whose activation by JNK and similar molecules promotes the expression of several inflammatory cytokines and leads to tissue damage (Figure 4) [146, 147].

The ameliorative effect of Sestrin2 on liver fibrosis was associated with the activation of AMPK signaling and downregulation of mTORC1/S6/4E-BP signaling [125, 142]. Previously, it was observed that the suppression of the mTORC1 signaling pathway is associated with apoptosis of activated hepatic stellate cells and protects against liver fibrosis [148]. It was shown that overexpression of Sestrin2 can prevent tunicamycin-induced ER stress-mediated hepatocyte death. Indeed, Sestrin2 negatively regulates C/EBP homologous protein (CHOP) to prevent ER stress-mediated apoptosis, and these effects were reversed in SESN2^+/-^ hepatocytes [125, 126]. In addition, as Sestrin2 can inhibit mTORC1 signaling, it can prevent excessive production of proteins and decrease cell susceptibility to ER stress and unfolded protein response [125]. Consistently, it was observed that Sestrin2 knockdown aggravated ER stress, and inhibition of mTORC1 by rapamycin could alleviate ER stress [149]. Interestingly, it was shown that during ER stress, protein kinase regulated by the RNA- (PKR-) like ER kinase (PERK)/CCAAT-enhancer-binding protein *β* (c/EBP*β*) axis induces Sestrin2 expression in hepatocytes (Figure 3) [125]. Kim et al. reported that during ER stress, the PERK/eukaryotic initiation factor-2*α* (eIF2*α*)/activating transcription factor 4 (ATF4) pathway can upregulate Sestrin2 expression in hepatocytes [124]. Jegal et al. also showed that activating transcription factor 6 (ATF6) can promote Sestrin2 expression to prevent ER stress-mediated apoptosis (Figure 3) [150]. Therefore, several members of ER stress can induce Sestrin2 expression, while Sestrin2 confines ER stress to prevent hepatocyte death.

In response to mitochondrial superoxide, Sestrin2, through ULK1, phosphorylates Beclin1 at the serine-14 position, preparing it for binding to Parkin. Sestrin2 potentiates the interaction between Beclin1 and Parkin leading to the translocation of the cytosolic Parkin to the mitochondrial surface. The translocation of Parkin leads to the mitophagy of defective mitochondria [100]. Sestrin2-mediated autophagy and mitophagy can improve mitochondrial dysfunction and prevent hepatocyte apoptosis [151].

The liver needs Sestrin2 to modulate cellular homeostasis, oxidative stress, autophagy, mitophagy, and apoptosis and regulate a wide variety of cellular signaling pathways. Therapeutic interventions to enhance the expression of Sestrin2 protects hepatocytes and can be a new candidate for the treatment of liver diseases.

## 4. Sestrin2 in Kidney Diseases

It was shown that Sestrin2 can protect against high-fat diet-associated renal fibrosis in rats. SESN2 deletion has been associated with podocyte loss and glomerular lesions in rats [152]. A significant increase in the serum levels of Sestrin2 was found in patients with diabetic nephropathy and metabolic syndrome [43]. Meanwhile, serum levels of Sestrin2 have been negatively associated with serum neutrophil gelatinase-associated lipocalin (NGAL) among healthy subjects and patients with type 2 diabetes with and without diabetic nephropathy [153]. Acute kidney injury, renal IR, and subsequent increase in oxidative stress activate the P53/Sestrin2 axis [154]. Sestrin2 plays a pivotal role in the attenuation of oxidative stress in acute kidney injury. Sestrin2 silencing augments oxidative stress and exacerbates kidney injury [60]. Sestrin2 expression is necessary for an appropriate response to stress conditions in the kidney, and insufficient Sestrin2 expression results in mTORC1 overactivity and, subsequently, apoptosis of renal cells. Downregulation of Sestrin2 in the kidney has been accompanied by glomerulosclerosis and severe periglomerular fibrosis in rats [155]. Sestrin2 overexpression alleviates renal I/R and increases autophagy and mitophagy in renal tubular cells [154]. Autophagy and mitophagy contribute to the removal of dysfunctional organelles and confine oxidative stress, ER stress, apoptosis, and necrosis in acute kidney injury and renal IRI [156]. Ineffective autophagy and mitophagy worsens tubular injury and renal function in acute kidney injury [156]. It was shown that Sestrin2 can attenuate ER stress in the kidney and prevent renal epithelial tubular cell EMT which is markedly involved in the progression of diabetic nephropathy [157].

Sestrin2 protects the kidney against oxidative stress by enhancing the Nrf2 function which simultaneously ameliorates hypertension [66]. In response to stress signals, Nrf2 upregulates the expression of several antioxidants such as glutathione, HO-1, glutathione S-transferase, and cytochromes to protect against AKI and CKD [158]. Sestrin2, a stress-inducible molecule, attempts to compensate for kidney injury and prevent renal fibrosis in patients with diabetic nephropathy [43].

## 5. Sestrin2 in the Nervous System

Higher oxygen consumption, higher abundance of polyunsaturated fatty acids, higher neuronal membrane surface area, using glutamate as a neurotransmitter, weak antioxidant defense, and several of other reasons make the nervous system more vulnerable to oxidative stress [159]. Microglial activation, mitochondrial dysfunction, and acceleration of oxidative stress are the leading causes of neuroinflammation that finally progresses to neurodegenerative diseases such as Alzheimer's disease, Parkinson's disease, and dementia [160].

### 5.1. Traumatic Brain Injury and Encephalitis

It was shown that the Sestrin2/Nrf2 pathway can improve traumatic brain injury by attenuating oxidative stress and apoptosis and decreasing brain edema and neurological deficit [161]. Luo et al. indicated that adenoassociated virus 2- (AAV2-) mediated Sestrin2 overexpression can improve sepsis-associated encephalitis in mice by enhancing AMPK/mTORC1/ULK1-dependent autophagy [17]. Sestrin2 also decreased apoptosis of hippocampal neurons; lowered tissue levels of inflammatory cytokines such as IL1*β*, IL6, and TNF-*α*; and improved loss of learning and memory function in the mice model of sepsis-associated encephalopathy [17]. Previously, it was shown that autophagy can prevent microglial activation during sepsis-associated encephalitis and decrease inflammatory cytokine release [162].

### 5.2. Stroke and Hypoxic-Ischemic Encephalopathy

Sestrin2 promotes M2 macrophage polarization and inhibits M1 macrophage polarization after cerebral ischemia [29]. Contrary to M2 macrophage polarization, M1 macrophage polarization exacerbates oxygen glucose deprivation-induced neuronal death after ischemic stroke [163]. Sestrin2 could enhance AMPK/PGC-1*α* to improve mitochondrial biogenesis in the rat model of cerebral I/R. Further, Sestrin2 downregulation aggravated oxidative stress, apoptosis, and neuronal damage and suppressed the AMPK/PGC-1*α* and Nrf2 signaling pathway [164]. Moreover, Sestrin2 could block P38 MAPK-mediated expression of inflammatory cytokines such as IL1*β*, IL6, IL18, and TNF-*α* in the cerebral I/R model [13].

Administration of human recombinant Sestrin2 improved neurologic function and restricted infarct size in the rat model of neonatal hypoxic-ischemic encephalopathy [165]. Sestrin2 overexpression decreased the increased permeability of the blood-brain barrier and alleviated brain infarct and edema in the rat model of neonatal hypoxic-ischemic encephalopathy induced by common carotid artery ligation followed by 150 minutes of hypoxia. Besides, it was shown that hypoxia-inducible factor 1-alpha (HIF-1*α*) increased Sestrin2 expression in severe hypoxia-ischemia. Subsequently, Sestrin2 inhibited HIF-1*α*-mediated vascular endothelial growth factor (VEGF) expression and decreased blood-brain barrier permeability within the first 24 hours after hypoxic-ischemic injury (Figure 3) [166]. Early-onset increase in VEGF expression leads to the disruption of the blood-brain barrier following ischemic stroke and allows inflammatory cell infiltration, causes vasogenic edema, and exacerbates neuroinflammation, whereas late-onset increase in VEGF can improve the outcome of ischemic stroke [167, 168]. Interestingly, Sestrin2, through an Nrf2/HO-1-dependent manner, could promote VEGF expression and angiogenesis in the rat model of ischemic stroke and decrease cerebral IRI, 5 and 10 days after ischemia [169, 170]. These findings show that Sestrin2 may improve cerebral IRI by preventing early-onset increase in VEGF, while augmenting late-onset increase in VEGF.

### 5.3. Spinal Cord Injury and Peripheral Nerve Damage

Wu et al. uncovered that the protective effect of brain-derived neurotrophic factor (BDNF) on 3-nitropropionic acid-induced neuronal damage is associated with increased expression of Sestrin2 and attenuation of oxidative stress. Furthermore, it was shown that the production of NO, formation of cGMP, and subsequent activation of cGMP-dependent protein kinase (PKG) are needed for BDNF-mediated Sestrin2 expression. The PKG, P50, and P65 subunits of NF-*κ*B form a complex that binds to the promoter of Sestrin2 and upregulates its expression [171]. These interactions can justify how stress signals upregulate Sestrin2 and how Sestrin2 modulates them.

Spinal cord injury is associated with ER stress. PERK/ATF4 activation after spinal cord injury increases Sestrin2 expression. Sestrin2 via AMPK/mTORC1 enhances autophagy and prevents apoptosis and unfolded protein response. Sestrin2 overexpression ameliorates ER stress, improves neuronal survival, and promotes functional recovery after spinal cord injury. Additionally, AMPK inhibition weakens the protective effects of Sestrin2 on spinal cord injury [172]. It was shown that Sestrin2 expression increases in the mice model of neuropathic pain. In addition, Sestrin2 knockout was associated with significantly increased late-phase neuropathic pain behavior and elevated ROS levels in the mice model of neuropathic pain. Furthermore, *tert*-butyl hydroperoxide, an ROS donor, stimulated prolonged pain behavior in naive SESN2^−/−^ mice [8].

### 5.4. Neurodegenerative Diseases like Alzheimer's Disease and Parkinson's Disease

The augmentation of AMPK has been proposed as a therapeutic mechanism for neurodegenerative diseases such as Parkinson's disease [173]. Meanwhile, increased oxidative stress has been vigorously implicated in the pathogenesis of neurodegenerative diseases [174]. Increased expression of Sestrin2 has been detected in the midbrain and serum of patients with Parkinson's disease [1, 175]. Additionally, it was shown that patients with Alzheimer's disease have higher serum protein and mRNA levels of Sestrin2 compared with the normal control group and patients with mild cognitive impairment. Patients with mild cognitive impairment also had higher serum levels of Sestrin2 compared with the normal control group [176]. Consistently, increased expression of Sestrin2 was observed in the animal model of Parkinson's diseases induced by 1-methyl-4-phenylpyridinium. Herein, 1-methyl-4-phenylpyridinium increased P53 expression, and subsequently, P53 provoked Sestrin2 expression [1]. SESN2 knockdown led to exacerbation of 1-methyl-4-phenylpyridinium-induced oxidative stress, mitochondrial dysfunction, and neuronal apoptosis [1]. Similarly, P53-mediated overexpression of Sestrin2 protected against sevoflurane-induced oxidative stress and apoptosis in neuronal cells [177]. Aging is accompanied by decreased expression of Sestrin2, and subsequently, a reduced capacity to compensate for neuronal damage and an increased risk of neurodegenerative diseases [178].

It was observed that amyloid *β* peptide accumulation stimulates Sestrin2 expression in primary cortical neurons. Downregulation of Sestrin2 decreased amyloid *β* peptide-mediated autophagy in neuronal cells, increased amyloid *β* peptide toxicity, and reduced cell survival [179]. It seems that Sestrin2 provides an endogenous protection against amyloid *β* peptide neurotoxicity, and downregulation of Sestrin2 exacerbates neuronal damage after amyloid *β* peptide exposure [179]. Sestrin2 can enhance the longevity of neuronal cells and increase their tolerance to endogenous or exogenous damages.

### 5.5. Ototoxicity and Retinal Damage

It was observed that cochlear Sestrin2 expression decreases during older age. In addition, SESN2 knockout increased macrophage infiltration into the cochlea and accelerated age-related cochlear degeneration [178]. Ebnoether et al. revealed that gentamycin-induced hair cell death is associated with downregulation of Sestrin2. Besides, Sestrin2 knockout was associated with greater hair cell loss after exposure to gentamycin [180]. The protective effect of gossypol acetic acid on the retinal pigmented layer was shown to be mediated through Sestrin2. Gossypol acetic acid led to Forkhead box O3 (FoxO3) nuclear translocation. Thereafter, FoxO3 promoted SESN2 transcription by binding to its enhancer [181]. Sestrin2 overexpression also protected against hydrogen peroxide-induced retinal ganglion cell damage by upregulating Nrf2. Consistently, inhibition of Nrf2 abrogated the protective effects of Sestrin2 on retinal ganglion cells [182].

Accumulating evidence shows that Sestrin2 can protect against several neurologic diseases. It acts through various mechanisms to exert its protective effects. Lower expression of Sestrin2 during senescence can contribute to the development of neurodegenerative diseases which can be a new therapeutic target.

## 6. Respiratory Diseases

### 6.1. Obstructive Sleep Apnea (OSA)

Higher rates of oxidative stress have been identified in patients with OSA. Moreover, oxidative stress positively correlates with the severity of OSA [183, 184]. It was shown that patients with OSA have significantly higher plasma and urinary levels of Sestrin2. Plasma and urinary Sestrin2 positively correlated with the apnea-hypopnea index (AHI) and the severity of OSA and negatively correlated with mean oxygen saturation. Furthermore, Sestrin2 levels significantly decreased after using continuous positive airway pressure (CPAP) for four weeks [185, 186]. The plasma level of Sestrin2 has been proposed as a diagnostic and prognostic marker for OSA. Chai et al. proposed the Sestrin2 plasma level of 1.86 ng/mL as a diagnostic cut-off value for OSA with a sensitivity of 81.58% and a specificity of 61.54%. In addition, 5.21 ng/mL plasma Sestrin2 has been proposed as a cut-off value for severe OSA with a sensitivity of 61.90% and a specificity of 90.70% [187]. OSA is also complicated with several metabolic complications such as cardiovascular diseases in which Sestrin2 plays an ameliorative role [188]. Due to increased oxidative stress in patients with OSA, Sestrin2 has higher expression in these patients and protects them against cellular damage. Sestrin2 possesses a diagnostic and prognostic value and can be used for better management of OSA.

### 6.2. COPD and Asthma

Increased oxidative stress is a contributory factor for COPD and asthma which augments inflammation by activating NF-*κ*B and P38-MAPK, increases airway hyperresponsiveness, accelerates TGF-*β*-mediated fibrogenesis and airway remodeling, facilitates aging of the lungs and carcinogenesis, and enhances steroid resistance [189, 190].

It was reported that both serum level and tissue expression of Sestrin2 are higher in patients with COPD. Furthermore, a positive correlation was found between serum Sestrin2 level and serum MMP9 level or airway remodeling in chest CT-scan [191]. Similarly, it was uncovered that patients with asthma have higher plasma levels of Sestrin2. A negative correlation was found between plasma levels of Sestrin2 and FEV_1_% predicted and FEV_1_/FVC ratio during and after exacerbation of asthma [192]. In addition, higher levels of Sestrin2 have been identified in the sputum samples of patients with severe asthma, compared with patients with mild to moderate asthma [193].

It was observed that the anti-inflammatory effect of azithromycin on lung epithelial cells exposed to cigarette smoke extract is associated with upregulation of Sestrin2 [194]. Moreover, Sestrin2-mediated decrease in hyperoxidized peroxiredoxin could alleviate cigarette smoke-induced pulmonary alveolar type II epithelial cell injury [195]. In response to airway epithelial damage, HIF-1*α* promotes Sestrin2 expression, thereby confining epithelial barrier dysfunction [196].

Sestrin2 can lower inflammation and prevent fibrosis and airway remodeling in COPD. However, it was shown that attenuation of ROS, particularly superoxide anions through Sestrin2/Nrf2 signaling, can downregulate TGF-*β*/Smad and platelet-derived growth factor receptor-beta (PDGFR*β*) and enhance cigarette smoke-mediated emphysema in the mice model [197–199]. Likewise, Sestrin2 inactivation could partly prevent pulmonary emphysema in these mice [197, 198].

Impaired autophagy response, either decreased or increased, has been implicated in the pathogenesis of COPD [200]. It was revealed that ROS-induced overexpression of Sestrin2 increases autophagy by activating AMPK/mTORC1/ULK1 in the mice exposed to cigarette smoke [201]. It was shown that AMPK deficiency exacerbates cigarette smoke-mediated lung inflammation and emphysema in mice [202, 203]. In addition, Nrf2 activation could improve lung inflammation and emphysema, and Nrf2 deficiency was associated with more severe inflammation and emphysema in the mice model of cigarette smoke-induced lung inflammation [203, 204]. The available evidence is inconsistent towards the effect of Sestrin2 and its downstream molecules on COPD, and future studies are needed to shed light on this issue.

## 7. Sestrin2 Is an Autoregulatory Mechanism for Several Signaling Pathways

In this review, we have mentioned several interactions between Sestrin2 and other signaling pathways. It has been explained that some of these signaling pathways either protective or destructive can induce Sestrin2 expression. In return, Sestrin2 showed that it can modulate these signaling pathways to provide the utmost benefit for the damaged tissue. Herein, Nrf2 can enhance Sestrin2 expression, and Sestrin2 can activate Nrf2 by releasing it from Keap1 sequestration. In addition, it was discussed that Ang-II or ER stress can induce Sestrin2 expression by several signaling pathways such as JNK signaling pathway. In return, Sestrin2 can inhibit ER stress and the JNK signaling pathway and attenuate Ang-II-mediated effects. The same scenario was found to happen for HIF-1*α*, TGF-*β*, and NF-*κ*B. These findings show that Sestrin2 can provide an endogenous autoregulation mechanism for the proper function of the living cells. Sestrin2 can be induced by inflammation and damage signals to attenuate them. Besides, it can strengthen the protective mechanisms against tissue damage (Figure 3).

## 8. Can Sestrin2 Level Be Routinely Used for Clinical Purposes?

Sestrin2 can be routinely detected by serologic tests and available techniques such as the enzyme-linked immunosorbent assay (ELISA) method [25, 26]. However, western blotting, polymerase chain reaction (PCR), and other molecular tests are applicable for measuring the expression of Sesrin2 in tissue biopsies particularly in cancers [205, 206]. Sestrin2 can accelerate the diagnosis of several diseases and help to estimate their prognosis [25, 26]. For instance, it has been mentioned how Sestrin2 levels are associated with the presence or exacerbation of atherosclerosis, heart failure, myocardial infarction, diabetic nephropathy, and OSA in patients [25, 26, 153, 185]. Ye et al. found that Sestrin2 plasma levels are positively correlated with the angiographic atherosclerosis score known as the Gensini score [207]. The Sestrin2 level can even differentiate between several differential diagnoses [208]. Interestingly, it was shown that the pleural effusion Sestrin2 level can successfully distinguish between malignant pleural effusion and congestive heart failure, tuberculosis, and parapneumonic pleural effusion [208]. Yet, studies could not show a strong correlation between the levels of Sestrin2 and disease-specific biomarkers such as troponin, creatinine, cystatin C, and bilirubin. The functional role of Sestrin2 instead of being a structural protein and lack of tissue specificity can partly justify the absence of such correlation.

## 9. Conclusion and Future Direction

**Figure 5:**
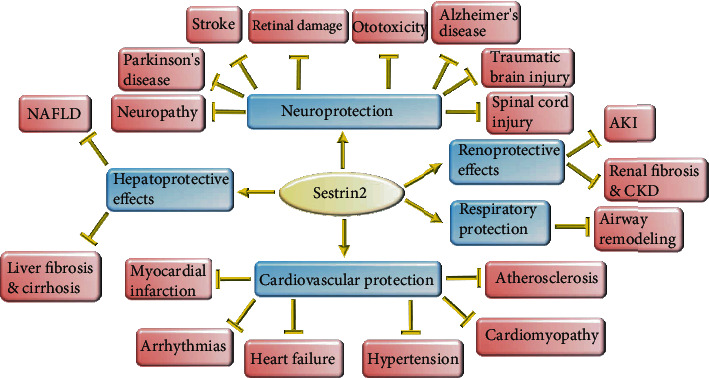
The protective effect of Sestrin2 on several organs. Sestrin2 can alleviate the diseases of several organs. It enhances the function of cardiovascular system, nervous system, respiratory system, liver, and kidney. It alleviates inflammation, prevents airway remodeling in the respiratory system, protects against liver and renal fibrosis, and mitigates NAFLD and AKI. Furthermore, Sestrin2 can profoundly improve cardiovascular and neuronal diseases. It ameliorates neuropathy, spinal cord injury, traumatic brain injury, retinal damage, hair cells damage, stroke, Parkinson's disease, and Alzheimer's disease. Sestrin2 can also enormously protect against myocardial infarction, arrhythmias, heart failure, atherosclerosis, hypertension, and cardiomyopathy.

Recent findings show that Sestrin2 is heavily involved in the pathogenesis of cardiovascular diseases, kidney diseases, liver diseases, respiratory diseases, and diseases of the nervous system and can improve the function of several organs (Figure 5). Primarily, Sestrin2 was considered just as an antioxidant; however, it was soon shown that Sestrin2 interacts with a wide variety of signaling pathways and regulates several cellular events such as autophagy, mitophagy, apoptosis, ER stress, mitochondrial biogenesis, lipogenesis, and fibrogenesis. Sestrin2 could enormously improve several diseases in vivo thanks to its vast molecular interactions. Pharmacological intervention to target Sestrin2 is rational in many chronic diseases and should be considered in future clinical trials.

## Data Availability

Data sharing is not applicable for this narrative review. No data were generated and analyzed for this article.
